# Prophylactic closed-incision negative pressure wound therapy for open ventral incisional hernia repair with mesh: a meta-analysis of randomized controlled trials

**DOI:** 10.3389/fsurg.2026.1962267

**Published:** 2026-10-19

**Authors:** Yihang Liu, Yuqing Lu, Song Li, Kaichun Li, Ming Li

**Affiliations:** 1 Department of General Surgery, Kaifeng155 Hospital, China RongTong Medical Healthcare Group Co.Ltd., Kaifeng, China; 2 Department of Gastroenterology, Huaihe Hospital, Henan University, Kaifeng, Henan, China; 3 Department of Urology, Kaifeng155 Hospital, China RongTong Medical Healthcare Group Co.Ltd., Kaifeng, China; 4 Kaifeng Minimally Invasive Diagnosis and Treatment Engineering Technology Research Center for Prostate Hyperplasia, Kaifeng, China; 5 Henan Province Prostate Disease Precision Diagnosis and Treatment Engineering Research Center of Traditional Chinese and Western Medicine, Kaifeng, China; 6 Discipline Construction Department, China RongTong Medical Healthcare Group Co.Ltd., Chengdu, China

**Keywords:** closed-incision negative pressure wound therapy, hernia repair, meta-analysis, surgical site infection, ventral incisional hernia

## Abstract

**Objective:**

To systematically evaluate the effects of prophylactic closed-incision negative pressure wound therapy (ciNPWT) on surgical site infection (SSI), seroma incidence and length of hospital stay after open ventral incisional hernia repair with mesh.

**Methods:**

Four databases (Cochrane Library, Embase, PubMed, Web of Science) were searched from inception to June 2026 for randomized controlled trials (RCTs) comparing prophylactic ciNPWT versus conventional sterile dressings in open ventral incisional hernia repair with mesh. The primary outcome was postoperative SSI incidence; secondary outcomes included seroma incidence and hospital stay. Study quality was assessed using the Cochrane Risk of Bias tool (RoB 1.0), and meta-analysis was performed with both fixed-effect and random-effects models using RevMan software. The certainty of evidence was assessed using the GRADE approach.

**Results:**

Four RCTs involving 420 patients (208 in ciNPWT group, 212 in control group) were included. Under the fixed-effect model, ciNPWT tended to reduce SSI risk without statistical significance (*RR* = 0.52, 95% CI 0.26–1.04, *P* = 0.06, *I^2^* = 50%). The random-effects model yielded a consistent but more conservative estimate (*RR* = 0.55, 95% CI 0.20–1.53, *P* = 0.25). No significant difference was found in seroma incidence (*RR* = 0.83, 95% CI 0.39–1.74, *P* = 0.61). ciNPWT was associated with shortened postoperative hospital stay (MD = −1.02 days, 95% CI −1.61 to −0.43, *P* = 0.0007), though this finding was based on only two studies and driven primarily by a single trial. Sensitivity analysis indicated that the SSI result was materially influenced by a single trial. Given the small number of included studies (k = 4), funnel plot asymmetry could not be reliably interpreted as evidence of publication bias. The GRADE certainty of evidence was rated as low for SSI and very low for seroma and hospital stay.

**Conclusion:**

Current evidence from randomized controlled trials suggests that prophylactic ciNPWT may be associated with shortened hospital stay after mesh-based ventral incisional hernia repair and shows a non-significant trend toward reducing SSI and seroma. However, the certainty of evidence is low to very low, the SSI result is materially dependent on a single trial, and the hospital stay finding is inconsistent across studies. Therefore, no definitive conclusion can be drawn regarding infection reduction, and the hospital stay benefit requires confirmation. More large-sample, multicenter, high-quality RCTs are needed for further validation.

## 1 Introduction

Ventral incisional hernia is a common disease in general surgery. As a long-term complication of abdominal surgery, incisional hernias have an overall incidence of 2%–20% and can significantly impair patients' postoperative quality of life (1, 2). Open mesh repair is currently the mainstream procedure for medium-to-large ventral incisional hernias. By implanting a synthetic mesh to achieve tension-free repair, it can effectively reduce the hernia recurrence rate (3). However, postoperative complications such as surgical site infection (SSI), seroma, and impaired wound healing remain core clinical challenges (4).

SSI is one of the most serious complications after mesh repair. Once it occurs, it not only prolongs hospital stay and increases medical burden, but may also progress to mesh infection in severe cases, leading to surgical failure and even requiring reoperation for mesh removal, imposing heavy physical, psychological, and economic burdens on patients (5). Seroma, the most common early postoperative complication, can cause local distending pain and incision exudation, and is also an independent risk factor for SSI (6, 7). Therefore, exploring safe and effective postoperative incision management strategies to reduce perioperative complication rates has long been an important research direction in abdominal wall hernia surgery (8).

Conventional sterile dressing coverage is the most basic postoperative incision care modality in clinical practice, but its effects are limited in reducing incision exudation, eliminating subcutaneous dead space, and improving the local microenvironment. Closed-incision negative pressure wound therapy (ciNPWT) is a prophylactic negative pressure technique developed for primarily closed incisions. By applying uniform and controllable negative pressure to the surface of a closed incision, it promotes local tissue blood circulation, reduces tissue edema, adequately drains perincisional exudate, and reduces subcutaneous dead space, theoretically lowering the risks of postoperative infection and seroma (9, 10).

In recent years, this technique has been gradually applied to high-risk incisions in multiple surgical fields, including orthopedics, general surgery, and obstetrics and gynecology. However, its clinical value in ventral incisional hernia repair with mesh remains controversial: some randomized controlled studies have shown that prophylactic ciNPWT can significantly reduce the incidence of postoperative SSI, while others have found no significant benefit compared with conventional dressings (11, 12). Moreover, there are considerable differences in negative pressure device types, treatment durations, and patient baseline characteristics across studies, limiting the generalizability of conclusions. Currently published systematic reviews on this topic have mostly included both randomized and non-randomized studies, resulting in high evidence heterogeneity, and pooled analyses of secondary outcomes such as seroma and length of hospital stay have been insufficient (13).

Accordingly, the present study strictly restricted inclusion to randomized controlled trials and comprehensively evaluated the effects of prophylactic closed-incision negative pressure wound therapy on SSI, seroma incidence, and length of hospital stay after open ventral incisional hernia repair with mesh through systematic review and meta-analysis, aiming to provide higher-level evidence-based medical evidence for the selection of clinical postoperative incision management strategies.

## 2 Methods

### 2.1 Search strategy

This systematic review and meta-analysis was conducted and reported in strict accordance with PRISMA 2020. The flow diagram is Figure 1. Four databases—the Cochrane Library, Embase, PubMed, and Web of Science—were electronically searched from inception to June 2026. Reference lists of included studies and reviews were manually traced; no additional studies were identified. The review protocol was not prospectively registered in a public registry (e.g., PROSPERO) prior to commencement, which is acknowledged as a methodological limitation.

**Figure 1:**
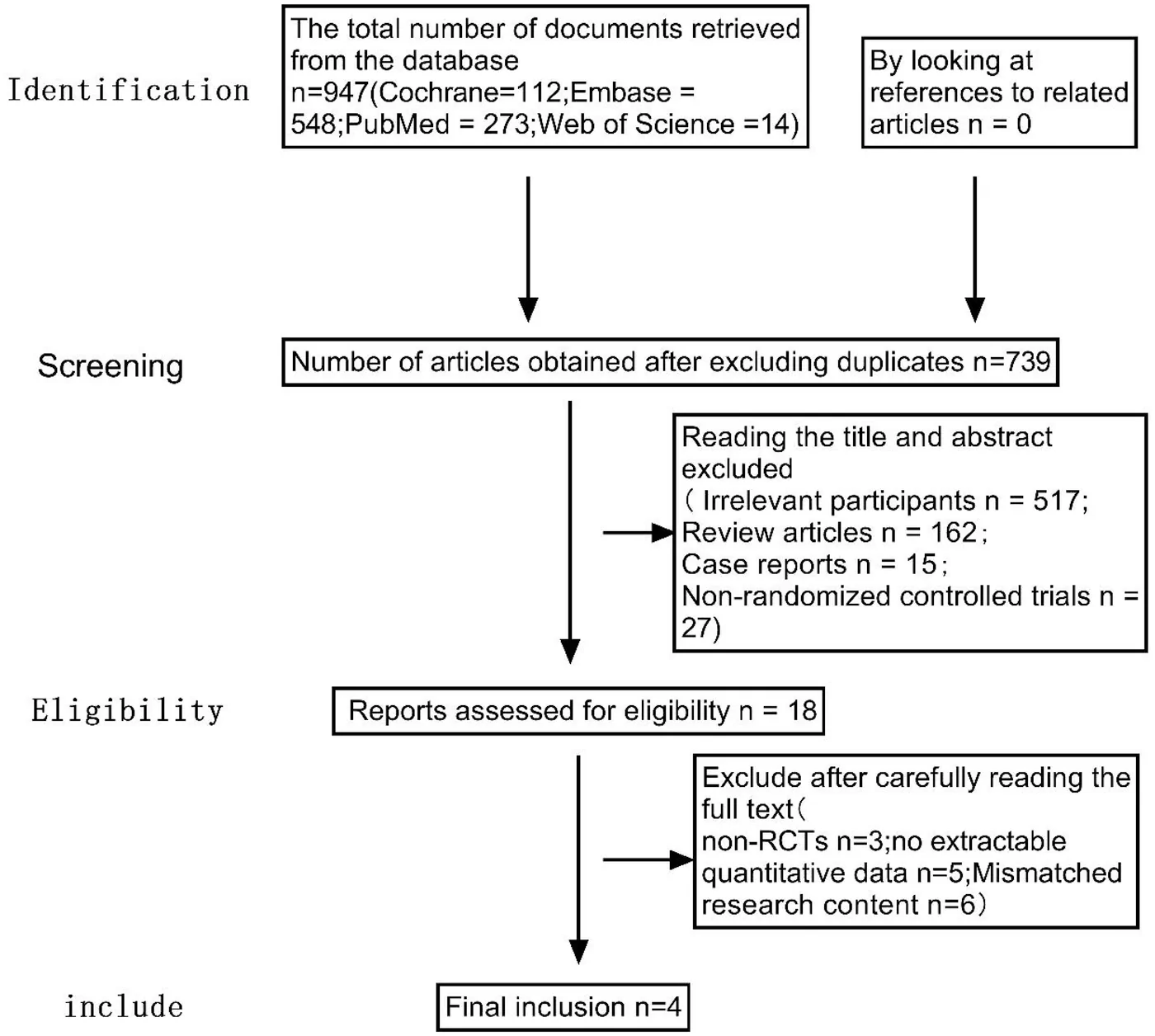
PRISMA 2020 flow diagram of literature screening.

The search strategy combined MeSH and free-text terms across three dimensions: intervention, disease, and study type. Complete reproducible search strings for each database are provided below.

PubMed: ((“Hernia, Ventral”[Mesh]) AND (“Negative-Pressure Wound Therapy”[Mesh])) OR (((npwt OR “negative pressure wound therapy” OR prevena OR pico OR VAC OR “vacuum assisted closure” OR ciNPWT OR “incisional negative pressure”) AND (ventral OR umbilical OR epigastric* OR incision* OR incisional) AND hernia AND (randomized controlled trial[ptyp] OR RCT[ti,ab])))
Embase: (“negative pressure wound therapy”/exp OR NPWT:ti,ab,kw OR PREVENA:ti,ab,kw OR PICO:ti,ab,kw OR ciNPWT:ti,ab,kw) AND (“Hernia, ventral”/exp OR “Hernia, incisional”/exp OR hernia:ti,ab,kw) AND (“ventral hernia repair”:ti,ab,kw OR VHR:ti,ab,kw) AND [randomized controlled trial]/lim
Cochrane: (npwt OR “negative pressure wound therapy” OR prevena OR pico OR ciNPWT):ti,ab,kw AND (hernia OR “ventral hernia” OR “incisional hernia”):ti,ab,kw AND (“ventral hernia repair” OR VHR OR “incisional hernia repair”):ti,ab,kw
Web of Science: TS = (npwt OR “negative pressure wound therapy” OR prevena OR pico OR ciNPWT) AND TS = (hernia OR “ventral hernia” OR “incisional hernia”) AND TS = (“ventral hernia repair” OR VHR OR “incisional hernia repair” OR IHR)

### 2.2 Literature screening and data extraction

Two researchers independently completed literature screening according to predefined inclusion and exclusion criteria. Preliminary screening was performed by reading titles and abstracts to exclude obviously irrelevant literature, followed by full-text review for final eligibility determination. Disagreements during the screening process were resolved through discussion and consensus; if consensus could not be reached, a third senior researcher made the final adjudication.

Data were extracted from included studies: first author, year, country, sample size, baseline characteristics (sex, age, BMI, ASA, comorbidities), hernia characteristics (defect size, EHS classification), surgical details (repair technique, mesh type, mesh position, operative time), intervention details (device brand/model, negative pressure setting, treatment duration, dressing protocol), SSI definition, follow-up duration, analysis set (ITT vs. available-case/per-protocol), and outcome data. Where both ITT and per-protocol were reported, modified ITT/available-case estimates were preferentially extracted. Cross-checking ensured accuracy.

### 2.3 Inclusion and exclusion criteria

#### 2.3.1 Inclusion criteria

1. Study type: Full-text randomized controlled trials (RCTs) published in English, with no restriction on blinding method.
2. Study population: Adult patients (aged ≥ 18 years) undergoing open ventral incisional hernia repair with mesh, with no restrictions on sex, hernia defect size, mesh material, or surgical reconstruction technique.
3. Interventions: The experimental group received prophylactic closed-incision negative pressure wound therapy immediately after primary skin closure (including various commercial or custom-made ciNPWT, sNPWT, INPWT and other closed-incision negative pressure systems). The control group received standard wound care with conventional sterile dressings (CWC).
4. Outcome data: At least one predefined outcome indicator was reported, with corresponding quantitative data and sample sizes extractable.

#### 2.3.2 Exclusion criteria

1. Non-randomized controlled study designs, including retrospective cohort studies, case series, case reports, conference abstracts, narrative reviews, and expert consensus.
2. Study populations with non-ventral incisional hernia types such as inguinal hernia or femoral hernia, or hernia repairs without mesh.
3. Negative pressure wound therapy applied to open wounds, established surgical site infections, or existing wound complications, rather than prophylactic use on primarily closed incisions.
4. No available quantitative outcome data, and valid data cannot be obtained after contacting the corresponding author.
5. Duplicate publications; in cases of overlapping data, only the version with the most complete data and longest follow-up duration was retained.
6. Studies with major design flaws, severe internal data contradictions, or clear factual errors.

### 2.4 Outcome measures

#### 2.4.1 Primary outcome

The primary outcome was the incidence of surgical site infection during follow-up, defined according to the criteria of the U.S. Centers for Disease Control and Prevention (CDC) and encompassing superficial incisional infection, deep incisional infection, and organ/space infection.

#### 2.4.2 Secondary outcomes

Incidence of postoperative seroma: Seroma confirmed by clinical examination or abdominal imaging during postoperative follow-up.

Postoperative length of hospital stay: Defined as the total number of days from the day of surgery to the day of discharge, expressed as mean ± standard deviation.

### 2.5 Methodological quality assessment

Two trained researchers independently assessed the methodological quality and risk of bias of the included randomized controlled trials using the Cochrane Risk of Bias tool (Version 1.0, RoB 1.0). Disagreements during assessment were resolved through discussion and consensus; if consensus could not be reached, a third senior researcher made the final adjudication.

The assessment covered seven predefined domains: (1) random sequence generation; (2) allocation concealment; (3) blinding of participants and personnel; (4) blinding of outcome assessment; (5) handling of incomplete outcome data; (6) selective outcome reporting; (7) other potential biases. Each domain was judged as low risk of bias, high risk of bias, or unclear risk of bias. The assessment results were finally summarized and presented as a risk of bias summary graph and a risk of bias bar graph.

### 2.6 Statistical analysis

Meta-analysis was performed using RevMan 5.4. For dichotomous outcomes (SSI, seroma), RR with 95% CI was used. For continuous outcomes (hospital stay), MD with 95% CI was calculated. Given moderate heterogeneity for SSI (*I^2^* = 50%), both fixed-effect (Mantel-Haenszel) and random-effects (DerSimonian-Laird) models were applied. For I^2^ < 25%, fixed-effect was primary. Heterogeneity was quantified by I^2^ and Cochran Q. Leave-one-out sensitivity analysis was performed for SSI. With k = 4, quantitative publication bias assessment was not feasible.

### 2.7 Certainty of evidence assessment (GRADE)

Certainty of evidence was assessed using the GRADE framework. RCT evidence starts at high certainty and may be downgraded for risk of bias, inconsistency, indirectness, imprecision, and publication bias. Certainty was rated high, moderate, low, or very low. A Summary of Findings table presents estimates alongside GRADE ratings.

## 3 Results

### 3.1 Flow chart of literature screening

Literature screening was performed strictly in accordance with PRISMA guidelines, and the screening process is shown in Figure 1. In the initial search, a total of 947 records were retrieved from the four databases (Cochrane Library: 112; Embase: 548; PubMed: 273; Web of Science: 14). No additional records were identified through manual reference tracing. After removing duplicates, 739 records remained for title and abstract screening. During this stage, 721 records were excluded, including 517 with irrelevant study populations, 162 review articles, 15 case reports, and 27 non-randomized controlled trials. The remaining 18 records underwent full-text eligibility assessment. After careful full-text review, 14 further records were excluded: 3 non-randomized controlled trials, 5 with no extractable quantitative data, and 6 with mismatched research content. After sequential screening, a total of 4 randomized controlled trials meeting all inclusion and exclusion criteria were finally included for meta-analysis. This flow diagram follows the four-phase standard structure of the PRISMA 2020 statement, including identification, screening, eligibility, and inclusion.

### 3.2 Methodological quality assessment of included studies

The quality of the 4 included RCTs was assessed using the Cochrane Risk of Bias tool (Figures 2, 3). Three studies (Marckmann 2025, Mondal 2022, Bueno-Lledó 2020) were at low risk for random sequence generation and allocation concealment (computer-generated randomization, sealed envelopes). However, Jelinek 2024 allocated patients by even/odd numbering (quasi-randomization) rather than true randomization, and did not report adequate allocation concealment; it was therefore classified as high risk for random sequence generation and unclear for allocation concealment. All 4 studies were low risk for incomplete data and selective reporting. Blinding of participants and surgeons was not feasible (high performance bias in all). Detection bias: 1 study high risk, 3 low risk. Other biases: unclear in all.

**Figure 2:**
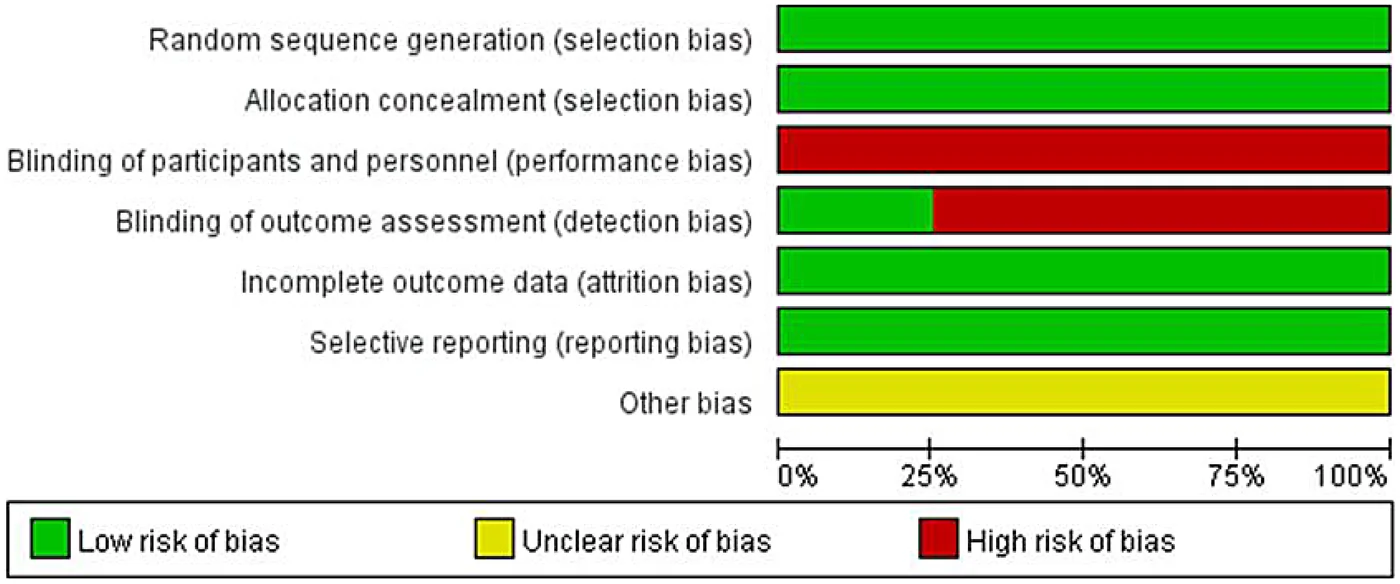
Risk of bias summary of included studies.

**Figure 3:**
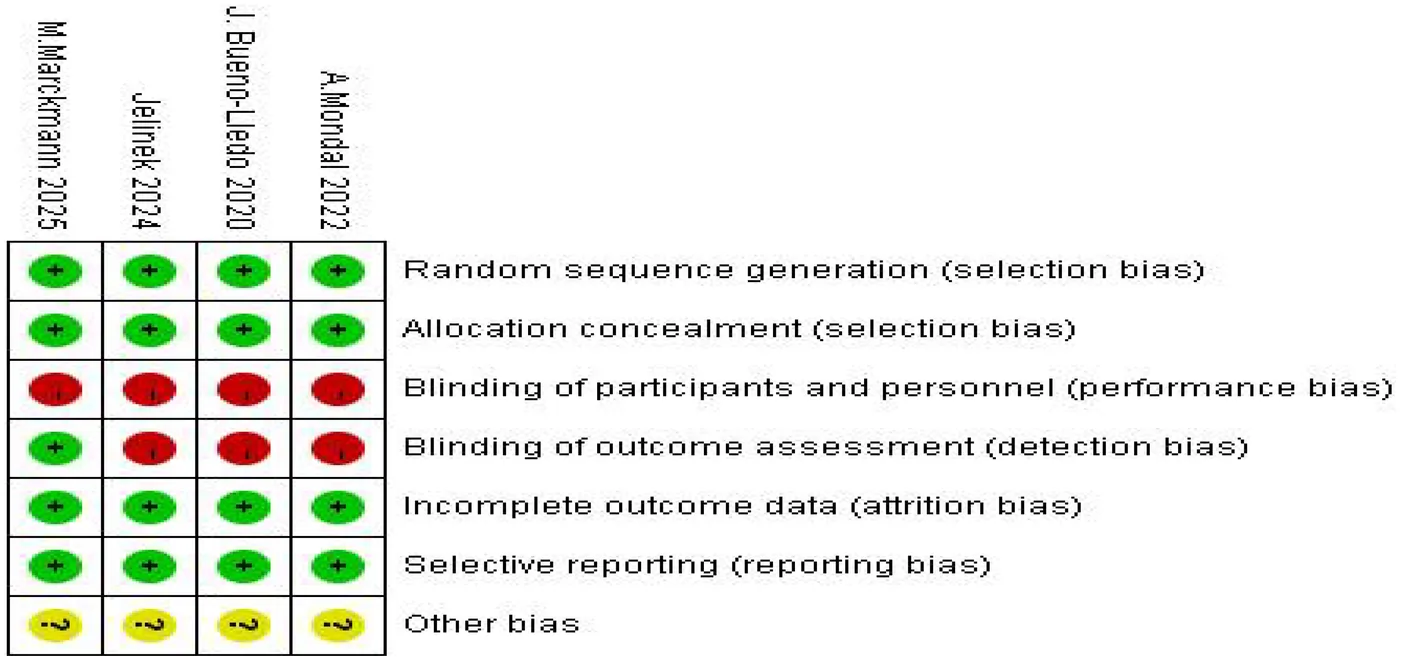
Risk of bias graph of included studies.

Overall, the methodological quality was moderate. Key limitations: (1) high performance bias in all studies due to inability to blind; (2) quasi-random allocation and unclear concealment in Jelinek 2024 (potential selection bias). These are reflected in GRADE downgrading.

### 3.3 Baseline characteristics of included studies

The baseline and clinical characteristics of the 4 included RCTs are summarized in Table 1. Studies were conducted in the Czech Republic, Denmark, India, and Spain (2020–2025), with sample sizes 64–146. All enrolled adults undergoing open ventral incisional hernia repair with mesh. Baseline characteristics (sex, age, BMI, operative time) were generally balanced between groups.

**Table 1:** Baseline characteristics of the included randomized controlled trials.

Study, Year, Country	Sample (N/C)	Age N/C	BMI N/C	Device/Brand	Pressure	Duration	Repair	Mesh	Follow-up
Jelinek 2024 (14) Czech	50/50	27–70/27–67	NR	Vivano® ciNPWT (HARTMANN)	−90 mmHg	5d	Onlay	Polypropylene	1y
Marckmann 2025 (15) Denmark	56/54	62.3 ± 11/60.3 ± 12	29.1 ± 4/29.6 ± 4	ciNPT (Acelity/KCI)	125 mmHg	POD3	RS/TAR/ACS	Polyprop/ composite	30d
Mondal 2022 (16) India	30/34	47 ± 12/43 ± 11	23.9 ± 3/23.8 ± 3	Custom INPWT (wall suction)	125 mmHg	5d	Onlay	Polyprop/ composite	4wk
Bueno-Lledó 2020 (17) Spain	72/74	51.6 ± 23/51.3 ± 19	NR (∼26% obese)	PICO® sNPWT (S&N)	−80 mmHg	7d	RS/TAR/ACS	Polyprop/ composite/ absorbable	30d

N/C, NPWT/control; RS, Rives-Stoppa; TAR, transversus abdominis release; ACS, anterior component separation; NR, not reported.

Negative pressure modalities varied: Vivano® ciNPWT (HARTMANN, −90 mmHg, 5 days), ciNPT (125 mmHg, POD 3 removal), custom INPWT (wall suction, 125 mmHg, 5 days), and PICO® sNPWT (Smith & Nephew, −80 mmHg, 7 days). Surgical techniques included Rives-Stoppa, TAR, and ACS, with polypropylene, composite, and absorbable meshes. There was notable heterogeneity in SSI definitions and follow-up: Marckmann 2025 and Bueno-Lledó 2020 used CDC criteria with 30-day follow-up; Mondal 2022 used CDC criteria with 4-week follow-up; Jelinek 2024 followed up to 1 year. These differences may have contributed to SSI heterogeneity.

### 3.4 Surgical site infection

For SSI, 4 RCTs (420 patients) were included. All reported data on an available-case or modified ITT basis (loss to follow-up 0%–1.8%). The Mantel-Haenszel fixed-effect model showed a trend toward SSI reduction (*RR* = 0.52, 95% CI 0.26–1.04, *P* = 0.06) with moderate heterogeneity (*I^2^* = 50%, *χ*^2^ = 6.04, *P* = 0.11) (Figure 4). Three studies favored NPWT, one (Marckmann) showed the opposite. No individual study was significant.

**Figure 4:**
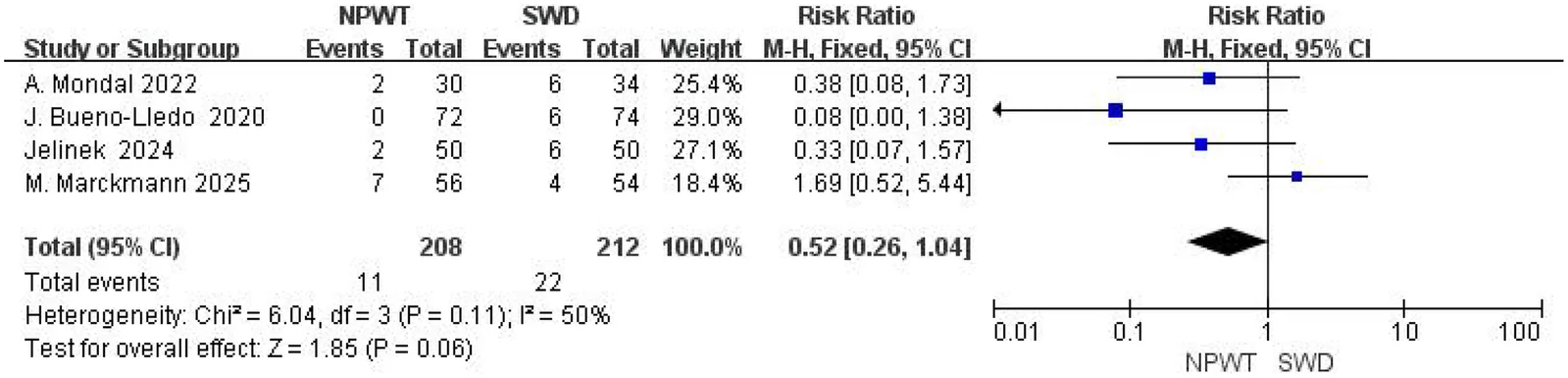
Forest plot of meta-analysis for the incidence of surgical site infection.

Given moderate heterogeneity (*I^2^* = 50%), a random-effects model (DerSimonian-Laird) was additionally applied: *RR* = 0.55 (95% CI 0.20–1.53, *P* = 0.25). The consistent but wider CI confirms that SSI reduction does not reach significance under either model, reflecting between-study variance and uncertainty.

### 3.5 Seroma

With postoperative seroma as the outcome, a total of 2 randomized controlled trials involving 210 patients were included (102 in the NPWT group and 108 in the conventional dressing group). The Mantel-Haenszel fixed-effects model was used to pool relative risks, and the results are shown in Figure 5. Heterogeneity testing indicated no heterogeneity among the included studies (*I^2^* = 0%, *χ*^2^ = 0.28, *P* = 0.60). The pooled effect size showed that the risk of postoperative seroma in the NPWT group tended to be lower than in the conventional dressing group, but the between-group difference did not reach statistical significance (*RR* = 0.83, 95% CI 0.39–1.74, Z = 0.50, *P* = 0.61). The conclusions of the two individual studies were generally consistent, both suggesting that NPWT slightly reduces seroma incidence. The 95% confidence intervals of both individual studies crossed the null value of 1, indicating no statistically significant within-study difference.

**Figure 5:**
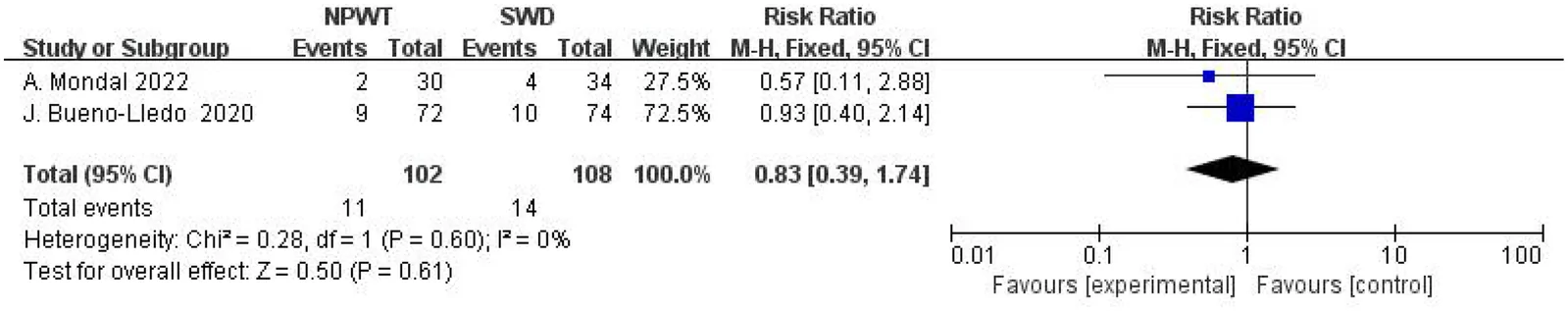
Forest plot of meta-analysis for the incidence of postoperative seroma.

### 3.6 Length of hospital stay

For hospital stay, 2 studies (210 patients) were included. Fixed-effect inverse variance: MD = −1.02 days (95% CI −1.61 to −0.43, *P* = 0.0007), *I^2^* = 0% (Figure 6). However, this was driven primarily by one study: Mondal 2022 showed borderline reduction (MD = −1.06, 95% CI −2.11 to −0.01, *P* = 0.057), while Bueno-Lledó 2020 showed non-significant reduction (MD = −0.30, 95% CI −1.01 to 0.41, *P* = 0.154). The third study (Jelinek 2024) found the opposite direction (ciNPWT associated with longer stay) but was not pooled (median, not mean). This inconsistency and only 2 contributing trials temper reliability. Initial stay should also be considered alongside readmissions, complications, and healthcare utilization, which were inconsistently reported.

**Figure 6:**
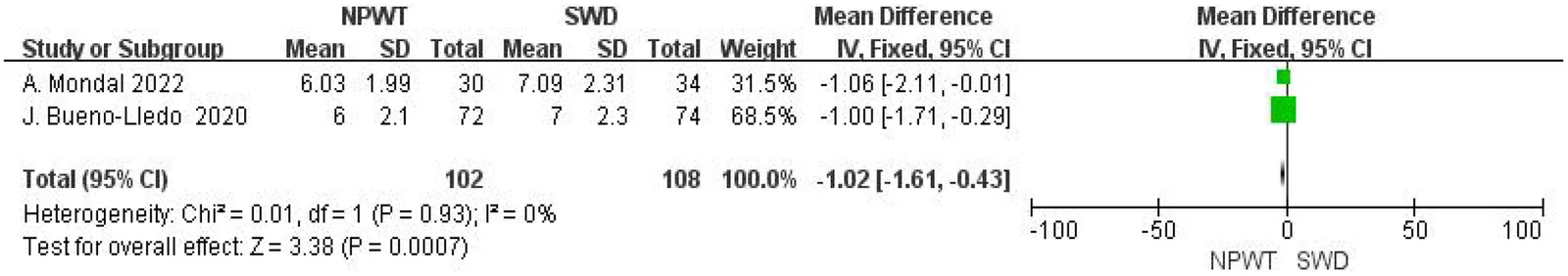
Forest plot of meta-analysis for postoperative length of hospital stay.

### 3.7 Sensitivity analysis

Leave-one-out sensitivity analysis was performed for the primary outcome of surgical site infection, with effect sizes re-pooled after sequential exclusion of each individual study. The results are summarized in Table 2.

**Table 2:** Leave-one-out sensitivity analysis for surgical site infection outcome.

Authors, Year	*P*	I^2^	RR[95%CI]	Test for overall effect
A. Mondal 2022 (16)	0.06	64	0.57 [0.26, 1.24]	0.16
J. Bueno-Lledo 2020 (17)	0.16	46	0.70 [0.33, 1.47]	0.35
Jelinek 2024 (14)	0.07	62	0.59 [0.27, 1.28]	0.18
M. Marckmann 2025 (15)	0.60	0	0.26 [0.09, 0.70]	0.008

The analysis showed that the study by M. Marckmann et al. published in 2025 was a high-influence study: after excluding this study alone, heterogeneity among the remaining 3 studies was completely eliminated (*I^2^* = 0%), the effect of NPWT in reducing SSI was significantly enhanced, the pooled RR decreased to 0.26 (95% CI 0.09–0.70), and the overall effect reached statistical significance (*P* = 0.008).

After individual exclusion of the other 3 studies, moderate heterogeneity remained among the remaining studies (*I^2^* = 46%–64%), and the 95% confidence intervals of the pooled RR all crossed the null value of 1, with overall effect *P* > 0.05. This was consistent with the core conclusion of the original meta-analysis—that NPWT reduces SSI but the difference is not statistically significant.

Overall, the SSI result showed marked instability, materially driven by the Marckmann trial. The fact that excluding a single trial reverses statistical significance indicates the evidence base is too sparse and fragile for a definitive claim. No definitive conclusion that NPWT reduces SSI can be drawn from current data.

### 3.8 Assessment of publication bias

Figure 7.

**Figure 7:**
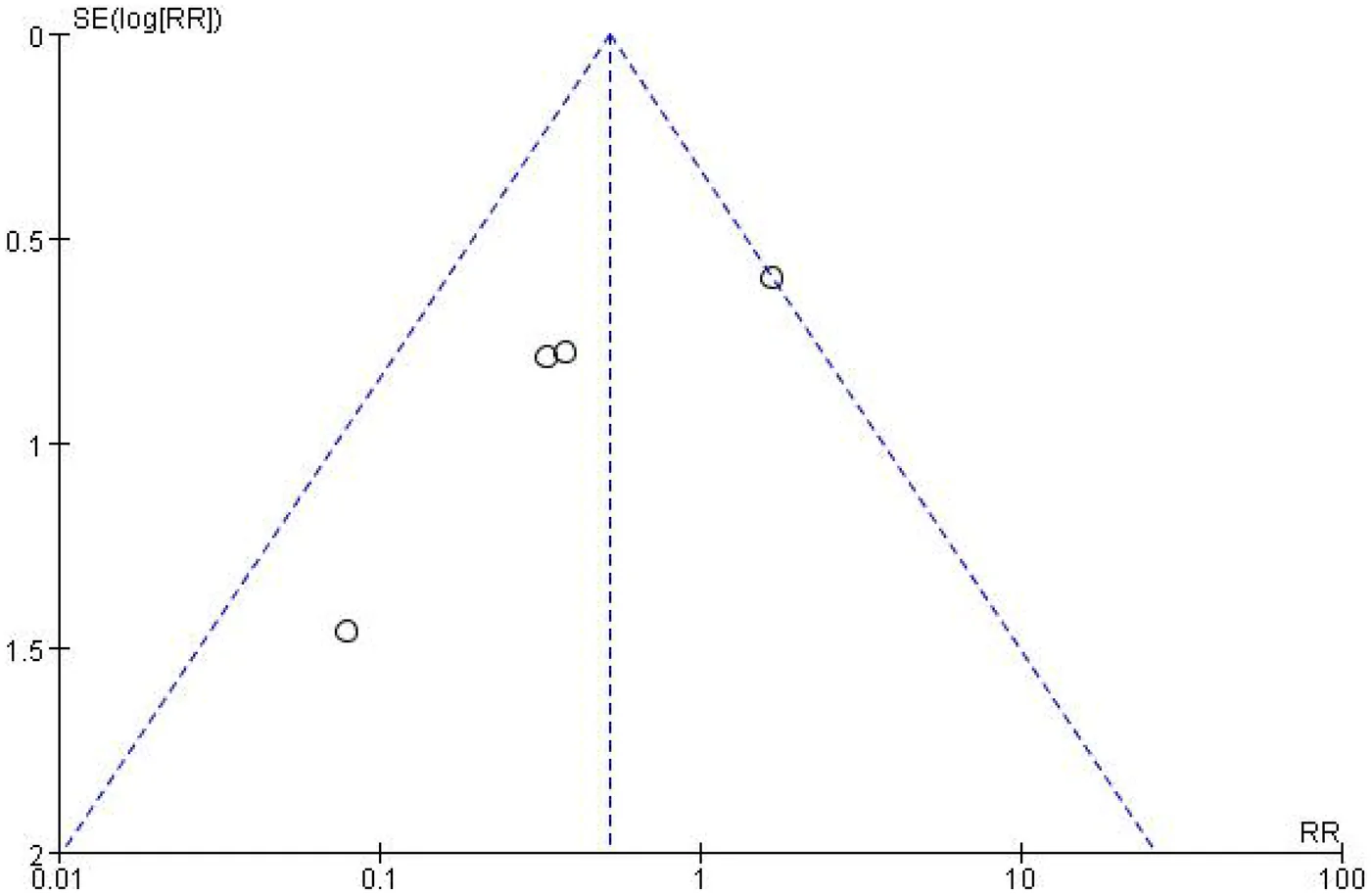
Funnel plot for surgical site infection outcome.

### 3.9 GRADE certainty of evidence

GRADE assessment (Table 3): SSI was downgraded two levels (−1 risk of bias: high performance bias + Jelinek quasi-randomization; −1 imprecision: wide CI, 33 events; moderate inconsistency *I^2^* = 50%, single-trial dependence) → Low. Seroma downgraded three levels (−1 risk of bias; −2 very serious imprecision: 2 studies, 25 events) → Very low. Hospital stay downgraded three levels (−1 risk of bias; −1 inconsistency: 3rd study opposite direction; −1 imprecision: 2 studies) → Very low.

**Table 3:** Summary of findings: GRADE certainty of evidence assessment.

Outcome	Effect (95% CI)	Downgrading	GRADE
SSI (4 RCTs, *n* = 420)	RR 0.52 (0.26–1.04) FE; RR 0.55 (0.20–1.53) RE	−1 RoB (performance bias, Jelinek quasi-random); −1 imprecision (33 events); moderate inconsistency	Low ⊕⊕⦻⦻
Seroma (2 RCTs, *n* = 210)	RR 0.83 (0.39–1.74)	−1 RoB; −2 very serious imprecision (2 studies, 25 events)	Very low ⊕⦻⦻⦻
Hospital stay (2 RCTs, *n* = 210)	MD −1.02 (−1.61 to −0.43)	−1 RoB; −1 inconsistency (3rd study opposite); −1 imprecision	Very low ⊕⦻⦻⦻

A funnel plot was constructed for SSI for descriptive purposes only (Figure 7). With only k = 4 studies, it has insufficient power to detect or rule out publication bias. Per the Cochrane Handbook, funnel plot tests are not recommended with fewer than 10 studies. Any asymmetry may reflect random variation or clinical heterogeneity, not publication bias. We do not interpret this plot as evidence of publication bias. The possibility of publication bias cannot be excluded or confirmed.

## 4 Discussion

This meta-analysis included 4 RCTs (420 patients) evaluating prophylactic ciNPWT in open ventral incisional hernia repair with mesh. ciNPWT was associated with significantly shortened hospital stay (pooled from 2 studies) and showed a non-significant trend for SSI and seroma. However, GRADE certainty was low for SSI and very low for seroma and hospital stay, indicating substantial uncertainty.

SSI can progress to mesh infection and surgical failure. ciNPWT theoretically lowers risk via microcirculation, edema reduction, exudate drainage, and tension reduction (18). The fixed-effect RR was 0.52 (95% CI 0.26–1.04, *P* = 0.06); random-effects RR was 0.55 (95% CI 0.20–1.53, *P* = 0.25). This should not be interpreted as a proven benefit, given the non-significant *P*-value, wider random-effects CI, and single-trial dependence. This differs from prior meta-analyses including non-randomized studies (11, 12, 19).

Reasons for the non-significant result: (1) strict RCT-only inclusion; (2) small sample (420 patients, 33 events), insufficient power; (3) moderate heterogeneity (*I^2^* = 50%) from differences in devices, pressure, duration, SSI definitions, and follow-up (20).

Sensitivity analysis showed that excluding Marckmann 2025 eliminated heterogeneity (*I^2^* = 0%) and rendered the result significant (*RR* = 0.26, *P* = 0.008). Reasons for this discrepancy are unclear and untested; potential factors (larger defects, obesity, dissection extent, stricter SSI criteria) are hypotheses requiring formal subgroup analyses. The dependence on a single trial demonstrates the evidence base is too fragile for definitive SSI claims.

Seroma is associated with defect size, dissection, and mesh reaction (18). Pooled *RR* = 0.83 (95% CI 0.39–1.74), non-significant. While ciNPWT drains subcutaneous exudate, deeper perimesh fluid cannot be drained (21). Only 2 studies contributed; no definitive conclusion.

Hospital stay pooled MD = −1.02 days (*P* = 0.0007), but driven primarily by Mondal (*P* = 0.057, borderline); Bueno-Lledó was non-significant (*P* = 0.154); Jelinek found opposite direction. GRADE certainty very low. Potential mechanisms include reduced exudation, fewer dressing changes, less pain, earlier ambulation. This finding is hypothesis-generating, not conclusive, and must be considered alongside readmissions and complications.

Limitations: (1) Only 4 RCTs (420 patients); SSI dependent on one trial. (2) Protocol not prospectively registered. (3) Publication bias unassessable (k = 4). (4) Clinical heterogeneity in devices (−80 to 125 mmHg), duration (3–7 days), SSI definitions, follow-up, techniques, meshes. (5) High performance bias (all); Jelinek quasi-randomization (selection bias). (6) English-only (language bias). (7) GRADE certainty low to very low.

In summary, current RCT evidence suggests ciNPWT may be associated with shortened hospital stay and shows a non-significant trend toward reducing SSI and seroma after mesh ventral incisional hernia repair. However, certainty is low to very low, SSI depends on a single trial, and hospital stay is inconsistent across studies. No definitive conclusion can be drawn regarding infection reduction, and hospital stay benefit requires confirmation. Statistical significance must be distinguished from clinical significance: even where significant (∼1 day stay), the absolute magnitude may be of limited clinical relevance versus device cost. The suggestion that ciNPWT might preferentially benefit high-risk subgroups (obesity, diabetes, large hernias) is biologically plausible but was not demonstrated (subgroup analyses not feasible); this is a hypothesis for future trials. ciNPWT use should be individualized. Future large multicenter RCTs with standardized protocols, blinded assessment, and prospective registration are needed.

## Data Availability

The original contributions presented in the study are included in the article, further inquiries can be directed to the corresponding author.

## Author disclaimer

The views and research conclusions presented in this paper regarding the efficacy of prophylactic closed-incision negative pressure wound therapy in open ventral incisional hernia repair with mesh are the independent academic insights of the authors derived from evidence-based medical analysis and research data. They solely reflect the authors' own positions and do not constitute the official views or formal stances of Huaihe Hospital of Henan University, Kaifeng 155 Hospital, or any other institutions affiliated with the authors.
